# Steroid-Resistant Nephrotic Syndrome Caused by *NUP93* Pathogenic Variants

**DOI:** 10.3390/jcm12185810

**Published:** 2023-09-07

**Authors:** Anna Wasilewska, Agnieszka Rybi-Szuminska, Pawel Dubiela

**Affiliations:** 1 Paediatric Nephrology Department, Medical University of Bialystok, Waszyngtona 17, 15-273 Białystok, Poland; anna.wasilewska@udsk.pl (A.W.); agnieszka.rybi@udsk.pl (A.R.-S.); 2 Department of Regenerative Medicine and Immune Regulation, Medical University of Bialystok, Waszyngtona 13, 15-269 Bialystok, Poland; 3 Department of Pathophysiology and Allergy Research, Medical University of Vienna, Waehringer Guertel 18-20, 1090 Vienna, Austria

**Keywords:** *NUP93*, SRNS, steroid-resistant nephrotic syndrome, paediatric nephrology, new pathogenic variant, nuclear pore complex proteins

## Abstract

Background: Although steroid therapy is a standard of care for nephrotic syndrome treatment, 15–20% of patients do not respond to it. Finding the genetic background is possible in >10% of steroid-resistant nephrotic syndrome (SRNS) cases. Variants in genes encoding nuclear pore complex proteins are a novel cause of paediatric steroid-resistant nephrotic syndrome (SRNS). Recent studies suggest *NUP93* variants to be a significant cause of paediatric onset SRNS. The clinical data on certain variants and disease history are still very limited. Methods and results: We report the SRNS case of a 12-year-old boy with two detected *NUP93* variants, which are pathogenic and possibly pathogenic. The onset of the disease was early and severe. The patient was admitted to the paediatric nephrology department due to nephrotic-range proteinuria and hypoalbuminemia with a long medical history of steroid and non-steroid immunosuppressive treatment. The genetic panel targeting 50 genes, clinically relevant for nephrotic syndrome, was performed. The only gene which was found to be affected by mutations, namely c.2326C>T and c.1162C>T, respectively, was *NUP93.* Conclusions: *NUP93* variants are rarely identified as causes of SRNS. Clinical data are of utmost importance to establish the standard of care for SRNS patients suffering from this genetic disfunction. This is the first case of a heterozygous patient with the c.2326C>T and c.1162C>T variants and confirmed clinical history of the SRNS described so far. Our data suggest the clinical relevance of the c.1162C>T variant.

## 1. Introduction

Nephrotic syndrome is a common chronic disorder with annual incidence ranging from 2 to 7 per 100,000 children, and a prevalence from 12 to 16 per 100,000 [1]. About 15–20% of patients with nephrotic syndrome are steroid-resistant (SRNS), suffering from proteinuria that does respond to a standard glucocorticoid treatment lasting four to six weeks. Nearly a half of these individuals achieve sustained remission after receiving an intensified immunosuppressive treatment. From the pathophysiological point of view, genetic and non-genetic cases of SRNS are defined as being or not being caused by pathogenic variants, respectively [2]. Seventy-nine genes were associated with SRNS and were suggested by International Paediatric Nephrology Association (IPNA) to be included in next-generation sequencing (NGS) in children with SRNS. Among them, there are seven nuclear-pore-associated genes: *NUP85*, *NUP107*, *NUP133*, *NUP160*, *NUP205*, *XP05* and *NUP93* [3].

Nucleoporins are essential components of the nuclear pore complex (NPC) [4]. Importantly, *NUP93*, expressed in all kidney cell types [5], is involved in NPC assembly, and its mutations are the direct cause of the complex disruption triggering a distinct renal disease [6]. To date, 21 different *NUP93* mutations are reported in 22 SRNS patients, with the most common variant, c.1772G>T, present in almost half of the described cases [7].

As defined by IPNA, the management of SRNS is a great challenge due to its heterogeneous ethology, patients’ responses to the therapy and safety complications including drug toxicity, infections, thrombosis, and the development of end-stage kidney disease (ESKD). One of the outlooks from recommendations is to perform genetic testing in all children diagnosed with primary SRNS. With this approach, it is possible to uncover a form of SRNS that is amenable to treatment (e.g., coenzyme Q10) or adopt the treatment accordingly [3]. Therefore, we here describe the case report of a patient with SRNS with two *NUP93* variants.

## 2. Case Synopsis

A twelve-year-old boy of Caucasian ethnicity presented in December 2022 with nephrotic syndrome, including nephrotic-range proteinuria (UPC of > 21.25 mg/mg), mild edema and hypoalbuminemia (<30 g/L). The initial serum creatinine level was 3.84 mg/dL, and the urea level was 123 mg/dL. Other pertinent laboratory evaluations at the time of presentation included an albumin level of 2.49 g/dL, a bicarbonate level of 20.6 mmol/L, a calcium level of 1.79 mmol/L, a phosphorus level of 4.76 mg/dL, a potassium level of 3.34 mmol/L, and a parathyroid hormone level of 103.1 pg/mL. Mild cardiomyopathy was detected with echocardiography. An ultrasound of the kidneys showed either diffuse echogenicity or loss of corticomedullary differentiation. The patient already suffered from stage 4 chronic kidney disease (eGFR 25.4 mL/1.73 m^2^/min), and at the time was being treated with prednisolone, mycophenolate mofetil, losartan and amlodipine. There is no positive renal family history in this patient’s case; both his parents and sister are healthy. Since he presented some respiratory infection symptoms, a nasal secretion sample was obtained, and the presence of respiratory syncytial virus (RSV) was confirmed.

During the patient’s initial visit to the hospital, his medical records from other hospitals were retrospectively screened, and it appeared that the condition started to occur in 2013 (at the age of 3) when the boy was hospitalized in Belarus. In the same hospital, a biopsy was performed in 2014, which revealed focal segmental glomerulosclerosis in one kidney, while in 2018 a similar tissue pathology was found in the other kidney after a sample biopsy. Afterwards, the boy was treated with four injections of rituximab and tacrolimus. The treatment in the hospital lasted until early December 2022.

To understand the background of the disease, a genetic panel was approached with 50 genes screened simultaneously via NGS. (*ACTN4*; *ADCK4*; *ANLN*; *APOL1*; *ARHGAP24*; *ARHGDIA*; *AVIL*; *CD2AP*; *COL4A3*; *COL4A4*; *COL4A5*; *COQ2*; *CRB2*; *DGKE*; *DLC1*; *EMP2*; *FAN1*; *FAT1*; *FN1*; *INF2*; *ITGA3*; *KANK1*; *KANK2*; *KANK4*; *LAMB2*; *LMX1B*; *MAFB*; *MAGI2*; *MYH9*; *MYO1E*; *NPHS1*; *NPHS2*; *NUP107*; *NUP133*; *NUP160*; *NUP205*; *NUP85*; *NUP93*; *OSGEP*; *PLCE1*; *PTPRO*; *SCARB2*; *SGPL1*; *SMARCAL1*; *TBC1D8B*; *TRPC6*; *TTC21B*; *WDR73*; *WT1*; *XPO5*). NGS was performed in certified laboratory. The sequences of the enriched DNA regions were analysed using a NovaSeq6000 sequencer (Illumina, San Diego, CA, USA). Genetic variants were identified using the Burrows–Wheeler Aligner. This test can detect 100% of substitutions and 95% of small deletions and insertions. The average depth of sequence coverage was 82.0, with a quality threshold of 99.4%. The assay included analysis of coding exon sequences along with 10–20 nucleotide intron flankers. The test confirmed two variants in *NUP93,* c.2326C>T, known as a pathogenic variant, and c.1162C>T, which is considered likely to be pathogenic. The results are presented in Table 1.

The initial treatment included infusions of 20% albumin, furosemide, pulses of methylprednisolone, parenteral antibiotic therapy, and metoprolol. Since renal disease progression was observed, with the creatinine level rising to 7.01 mg/dL, the urea level reaching 296 mg/dL, and finally eGFR dropping to 8.43 mL/1.73 m^2^/min, the decision was made to start continuous ambulatory peritoneal dialysis for 9 days, and then switching to continuous cyclic peritoneal dialysis. Control tests confirmed stabilizing renal function.

## 3. Discussion

Although the role of *NUP93* variants is attracting increasing interest and the evidence of its association with SRNS is growing, only a small number of pathogenic mutations in a limited number of patients have been identified to date. This, in turn, results in several limitations for clinicians dealing with patients that could carry these rare mutations. Our aim was to describe our patient with a confirmed clinical history and genetic results confirming two *NUP93* variants.

Our data are consistent with previous reports [5,6,7,9,10,11,12,13,14,15], highlighting *NUP93* variants as a cause of paediatric-onset SRNS. Interestingly, the individual described is heterozygous with two different variants. c.2326C>T is known as pathogenic, while c.1162C>T is classified as likely pathogenic. Considering the nature of the disease, in that it is autosomally recessive and requires two affected allele copies to be phenotypic, we can speculate that c.1162C>T is also pathogenic. However, heterozygous patients with only a single variant and clinical onset have been described by Bezdicka et al. [9], which limits our conclusion.

This case has partially confirmed a novel phenotype of extrarenal manifestations previously described in individuals with *NUP93* variants by Sandokji et al. [12]. The patient presented in this report also developed cardiomyopathy, but lacked neurological symptoms. Although case reports cannot lead to firm conclusions, this study provides is another argument confirming that *NUP93* variants may be responsible for a severe SRNS phenotype with other organs’ involvement.

Another lesson we might learn from this case comes from the retrospective analysis of the hospitalization in Belarus. Confirming steroid resistance in the beginning of the course of nephrotic syndrome could lead to escalation of immunosuppressive treatment. SRNS remains a challenge for paediatric nephrologists, and the treatment strategies are individualized and vary among physicians. For immune-mediated SRNS, the combination of a calcineurin inhibitors and corticosteroid may be considered as a major strategy. Rituximab’s timely administration with repeated dosing has been shown to induce favourable outcomes in SRNS. SRNS associated with coenzyme Q10-deficiency may benefit from CoQ10 supplementation [16]. The gathered knowledge from the described cases and case series highlights the need for diagnostic test availability and rapid action, as was also shown in our manuscript. Taking into consideration the disease progression and biopsy results, we believe that both treatment modification and genetic testing could be initiated earlier, as suggested by Trautmann et al. [3].

## 4. Conclusions

Since there is a lack of functional assays to identify SRNS patients and confirm the role of detected variants, all the detected mutations would be classified as variants of unknown significance. This leads to an important discussion about how to interpret the results of genetic testing in clinical practice relating to orphan diseases. Bearing in mind the rapid and increasing application of next-generation sequencing as a diagnostic tool in kidney medicine, and the limitations of available data, it is of utmost importance to describe case reports supporting laboratory findings. We believe our report adds another important piece to the puzzle.

## Figures and Tables

**Table 1:** Results of genetic testing. Variants detected in *NUP93*.

Gene	Variant	Localization	Amino Acid Affected	Homo/Heterozygote	Minor Allele Frequency in EU	ID dbSNP/ClinVar	Variant Pathogenicity Assessment
*NUP93*	ENST00000308159: c.2326C>T	exon_21/22	p.Arg776	Hetero	0.0000	rs768650810	Pathogenic
*NUP93*	ENST00000308159: c.1162C>T	Exon_11/22	p.Arg388Trp	Hetero	0.0024	rs145146218; 224968	Likely pathogenic *

* Likely pathogenic variants had a high probability of being pathogenic but without adequate evidence yet [8].

## Data Availability

More data are available upon request from the corresponding author.
